# A Pilot Study on the Association of Mitochondrial Oxygen Metabolism and Gas Exchange During Cardiopulmonary Exercise Testing: Is There a Mitochondrial Threshold?

**DOI:** 10.3389/fmed.2020.585462

**Published:** 2020-12-21

**Authors:** Philipp Baumbach, Christiane Schmidt-Winter, Jan Hoefer, Steffen Derlien, Norman Best, Marco Herbsleb, Sina M. Coldewey

**Affiliations:** ^1^Department of Anesthesiology and Intensive Care Medicine, Jena University Hospital, Jena, Germany; ^2^Septomics Research Center, Jena University Hospital, Jena, Germany; ^3^Institute of Physiotherapy, Jena University Hospital, Jena, Germany; ^4^Department of Sports Medicine and Health Promotion, Friedrich Schiller University, Jena, Germany; ^5^Center for Sepsis Control and Care, Jena University Hospital, Jena, Germany

**Keywords:** COMET, mitochondrial oxygen tension (mitoPO_2_), mitochondrial oxygen consumption (mitoV̇O_2_), cardiopulmonary exercise testing (CPET), ergospirometry, protoporphyrin-IX triplet state lifetime technique, mitochondrial oxygen delivery (mitoḊO_2_)

## Abstract

**Background:** Mitochondria are the key players in aerobic energy generation via oxidative phosphorylation. Consequently, mitochondrial function has implications on physical performance in health and disease ranging from high performance sports to critical illness. The protoporphyrin IX-triplet state lifetime technique (PpIX-TSLT) allows *in vivo* measurements of mitochondrial oxygen tension (mitoPO_2_). Hitherto, few data exist on the relation of mitochondrial oxygen metabolism and ergospirometry-derived variables during physical performance. This study investigates the association of mitochondrial oxygen metabolism with gas exchange and blood gas analysis variables assessed during cardiopulmonary exercise testing (CPET) in aerobic and anaerobic metabolic phases.

**Methods:** Seventeen volunteers underwent an exhaustive CPET (graded multistage protocol, 50 W/5 min increase), of which 14 were included in the analysis. At baseline and for every load level PpIX-TSLT-derived mitoPO_2_ measurements were performed every 10 s with 1 intermediate dynamic measurement to obtain mitochondrial oxygen consumption and delivery (mito$\dot{\text{V}}$O_2_, mito$\dot{\text{D}}$O_2_). In addition, variables of gas exchange and capillary blood gas analyses were obtained to determine ventilatory and lactate thresholds (VT, LT). Metabolic phases were defined in relation to VT1 and VT2 (aerobic: <VT1, aerobic-anaerobic transition: ≥VT1 and <VT2 and anaerobic: ≥VT2). We used linear mixed models to compare variables of PpIX-TSLT between metabolic phases and to analyze their associations with variables of gas exchange and capillary blood gas analyses.

**Results:** MitoPO_2_ increased from the aerobic to the aerobic-anaerobic phase followed by a subsequent decline. A mitoPO_2_ peak, termed mitochondrial threshold (MT), was observed in most subjects close to LT2. Mito$\dot{\text{D}}$O_2_ increased during CPET, while no changes in mito$\dot{\text{V}}$O_2_ were observed. MitoPO_2_ was negatively associated with partial pressure of end-tidal oxygen and capillary partial pressure of oxygen and positively associated with partial pressure of end-tidal carbon dioxide and capillary partial pressure of carbon dioxide. Mito$\dot{\text{D}}$O_2_ was associated with cardiovascular variables. We found no consistent association for mito$\dot{\text{V}}$O_2_.

**Conclusion:** Our results indicate an association between pulmonary respiration and cutaneous mitoPO_2_ during physical exercise. The observed mitochondrial threshold, coinciding with the metabolic transition from an aerobic to an anaerobic state, might be of importance in critical care as well as in sports medicine.

## Introduction

Mitochondria are the power houses of aerobic cells. Fueled by oxygen and energy-rich substrates of the glucose, fat, and protein metabolism, they generate ATP, the energy equivalents required for the work of muscles and function of organs. It is obvious that damage to these organelles leads to a reduced energy supply on a cellular level. Thus, mitochondria play a pivotal role in physiologic and pathophysiologic conditions. With a better understanding of the complex interactions in sepsis, microcirculation and mitochondrial dysfunction have become a major focus of research (1). Numerous sepsis-associated mitochondrial abnormalities have been described: disturbances of the electron transport chain and of oxidative phosphorylation, structural damage, oxidative and nitrosative stress, proton leak and uncoupling being only a small selection (2). In response to these findings, mitochondria targeted therapies are currently being developed (3).

However, a non-invasive diagnostic tool for the assessment of mitochondrial performance *in situ* that can be applied simultaneously with an event of interest, such as the onset of sepsis or other physiological or pathophysiological conditions, is as yet missing. Apart from few exceptions (2, 4), mitochondrial function has mostly been assessed by laboratory tests with vital cells following invasive measures on patients, such as blood withdrawal or muscle biopsy (4).

Since oxygen is crucial to achieve a sufficient energy supply by aerobic ATP generation, it seems logical to employ mitochondrial oxygen tension and its dynamics as a marker for mitochondrial performance. To enable direct and non-invasive measurements of mitochondrial oxygen metabolism, Mik et al. introduced the protoporphyrin IX-triplet state lifetime technique (PpIX-TSLT) (5). Recently, the measurement of mitochondrial oxygen tension (mitoPO_2_) in skin became clinically available by the COMET system (Photonics Healthcare BV, Utrecht, Netherlands). Using the same system, oxygen consumption (mito$\dot{\text{V}}$O_2_) (6) and oxygen delivery (mito$\dot{\text{D}}$O_2_) (7) can be determined. A detailed description of the device is provided by Ubbink et al. (8). Up to now, just a few *in vivo* studies have been conducted and even less in patients or volunteers (7, 9–12).

**Figure 1:**
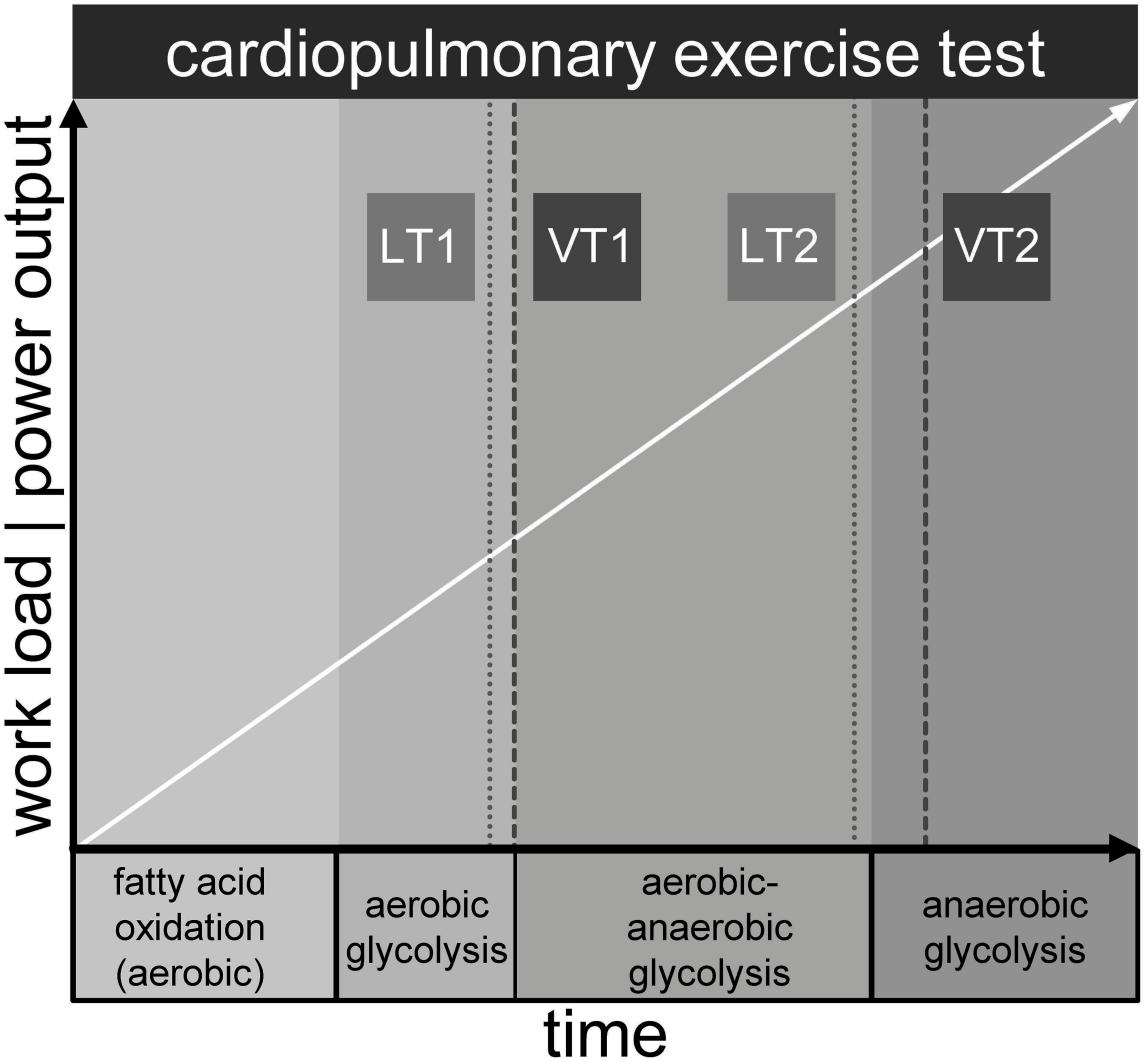
Metabolic phases and exemplary location of lactate (LT1, LT2) and ventilatory thresholds (VT1, VT2) during maximum cardiopulmonary exercise testing.

The OXPHOS complex on the inner mitochondrial membrane is the final acceptor of oxygen, which is provided by respiration and transported to tissue by blood, where it is bound to hemoglobin of the erythrocytes (13). While resting and during light physical activity, aerobic energy generation from fatty acids and in second line carbon hydrates, is the dominant feature in generating ATP. Yet, with increasing work intensity, oxygen demand is no longer balanced by supply. The consequence is additional allocation of ATP by anaerobic glycolysis. The buffering of H^+^ ions from the production of lactic acid yields extra CO_2_ that must be eliminated via enhanced respiration. This is the physiologic equivalent of the first ventilatory threshold (VT1) detected in ergospirometry (also known as cardiopulmonary exercise testing, CPET) (14). CPET from light to maximal intensity displays three phases: aerobic metabolism, aerobic-anaerobic transition, and anaerobic metabolism (15, 16). Ventilatory thresholds VT1 and VT2 separate the phases, whereas VT2 denotes the respiratory compensation point (RCP) (17). At this point respiration is intensified once more in order to counterbalance metabolic acidosis caused by a disproportionate lactic acid increase. Lactate levels can be measured directly in arterial or capillary blood samples. Similar to VT1 and VT2, two metabolic thresholds of the lactate kinetics (LT1 and LT2) can be identified. The latter are related to but not equal to the ventilatory thresholds (17). Of note, not less than 25 different LT concepts have been described (18). CPET measurements reveal a complex but not comprehensive picture of metabolic and respiratory processes during exercise (see Figure 1). The integration of the chronological sequences in the mitochondrion during exercise into the macroscopic observations of the CPET might not only be a useful contribution to the current knowledge of the complex processes of physical activity but also provide insights into mitochondrial performance when reaching physiologic or pathologic limitations.

In critical illness, the aerobic-anaerobic and the anaerobic metabolism are of utmost importance, since the balance between oxygen supply and oxygen demand is frequently disturbed (1). Aim of our pilot study (PpIX-TSLT measurements during CPET) was to elucidate, if there is an association of cellular oxygen metabolism with gas exchange parameters in distinct metabolic phases. Hitherto, this question has not been addressed but is of great interest when exploring any perturbances of mitochondrial function as is assumed to be a major feature in, e.g., sepsis. In order to gain insights into the interactions of the different levels from organ to cell to cellular organelles, such mechanisms need to be explored in healthy humans first. Therefore, our study was designed to create all three phases of physical exertion: aerobic metabolism, aerobic-anaerobic transition and anaerobic metabolism by employing a graded multistage protocol. To approach our research question, parameters of mitochondrial oxygen metabolism (mitoPO_2_, mito$\dot{\text{V}}$O_2_, mito$\dot{\text{D}}$O_2_) were measured in relation to respiratory gas exchange variables, lactate kinetics and other variables derived from capillary blood gas analyses during a maximal intensity CPET. We then analyzed the kinetics of mitoPO_2_, mito$\dot{\text{V}}$O_2_ and mito$\dot{\text{D}}$O_2_ in relation to the metabolic phases and to the metabolic (LT1 and LT2) and ventilatory thresholds (VT1 and VT2) (17).

## Methods

### Study Population

We recruited healthy male adults via the Jena University Hospital notice boards, notice boards in fitness-studios and social media. Participants had to be at least 18 years old, non-smoking, and should perform strength or fitness-oriented training at least three times a week. All candidates gave their written informed consent and according to the Physical Activity Readiness Questionnaire (19), showed readiness for physical activity, which the study team had to confirm. The following criteria lead to exclusion from participation: significant cardiac, pulmonary or musculoskeletal disease or condition, allergies to contents of the Alacare® plaster (Photonamic, Wedel, Germany), or to ingredients of Finalgon® CPD Wärmecreme (Sanofi Aventis, Germany), allergy to medical adhesive bandages, porphyria, skin conditions aggravated by sunlight or increased sensitivity to light, participation in another interventional study, or prior participation in this study. The study was approved by the ethics committee of the Friedrich Schiller University Jena (2019-1296-BO) and is registered at the German Clinical Trials Register (DRKS00016670).

### Experimental Setup of the Study

**Figure 2:**
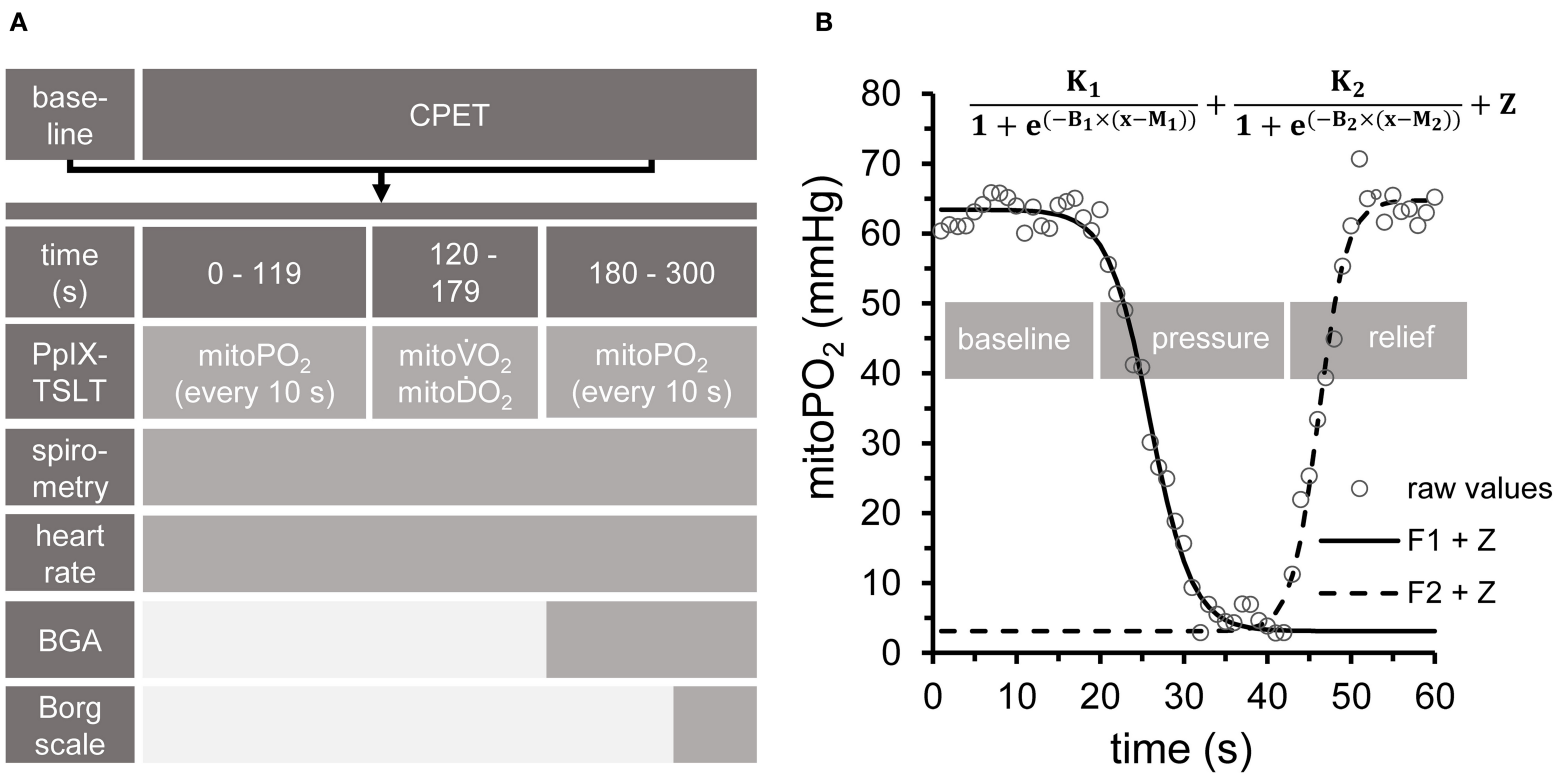
Experimental setup. **(A)** All measurements at baseline, during cardiopulmonary exercise testing (CPET) and in the recovery phase followed the same sequence. PpIX-TSLT measurements were performed every 10 s during the first and last 2 min of each phase to obtain mitochondrial oxygen tension (mitoPO_2_). Capillary blood samples for blood gas analysis (CBG) were taken after 180 s. Subjective ratings of exertion and dyspnea were assessed by the Borg Scale at the end of each phase. ECG and gas exchange parameters were monitored continuously. In addition, guideline-conform non-invasive blood pressure measurements were performed every 180 s. **(B)** After 2 min phase duration, dynamic PpIX-TSLT measurements were performed to assess mitochondrial oxygen consumption (mito$\dot{\text{V}}$O_2_) and delivery (mito$\dot{\text{D}}$O_2_). During the first 20 s, baseline mitoPO_2_ measurements were obtained. Thereafter, pressure was applied to the sensor in order to locally inhibit microcirculation. When mitoPO_2_ reached near to zero levels or showed no further decline, pressure was maintained for 10 s and then released. Based on the fitted parameters mito$\dot{\text{V}}$O_2_ (F1: left part of the equation) and mito$\dot{\text{D}}$O_2_ (F2: right part of the equation) were estimated [for details see (11)].

The complete setup is summarized in Figure 2.

#### PpIX-TSLT Measurements

This optical method uses an endogenous organic compound, protoporphyrin IX (PpIX), as a fluorophore. After excitation with a pulse of green light, it will emit a red delayed fluorescence (20). In the presence of oxygen, the lifetime of the delayed fluorescence caused by the first excited triplet state of porphyrin is inversely related to PO_2_ (20) as described by the Stern-Vollmer equation (21). The fact that oxygen is a quencher of fluorescence has been used to measure the oxygen content of mitochondria (5). Though PpIX, a natural precursor of heme in the heme biosynthetic pathway, is synthesized within the mitochondrion (22), its amounts are too small for the use as an oxygen-sensitive fluorophore. Because the conversion of PpIX to heme is a rate limiting step (23), its quantity can be enhanced by administering 5-aminolevulinic acid (5-ALA), a precursor of PpIX, to the measuring site (24). By pressure induced interruption of the local microcirculation oxygen consumption (mito$\dot{\text{V}}$O_2_) can be calculated from the rate at which the oxygen tension declines, while replenishment is stopped (6). Mitochondrial oxygen delivery (mito$\dot{\text{D}}$O_2_) however, is then calculated from the slope of the mitoPO_2_ increase, when pressure is released (11).

Individuals were instructed to apply a 4 cm^2^ adhesive patch containing 8 mg of 5-ALA (Alacare®, Photonamic, Wedel, Germany) to their left lumbar region at least 6 h prior to the measurement to ensure sufficient accumulation of PpIX. After application of Alacare®, the skin was protected from light with an adhesive plaster before and for 48 h after the test to prevent skin irritation. The lumbar region was chosen because of technical considerations: since the anterior chest wall, as proposed by Harms and colleagues (9), is not easily accessible and, in addition, is in constant movement while riding a bicycle, reliable measurements would not have been achieved. The measurements were performed with the COMET measurement system (Photonics Healthcare BV, Utrecht, Netherlands). After removal of the Alacare® plaster, the COMET Skin Sensor was fixed to the site with an adhesive tape to prevent shifting of the light source.

PpIX-TSLT measurements at baseline and during CPET always followed the same sequence (see Figures 2A,B). MitoPO_2_ was measured every 10 s at the beginning and at the end of each stage (baseline, each load level of CPET). After the second minute of each stage (to achieve steady state conditions), dynamic measurements (one measurement per second) were done to obtain mito$\dot{\text{V}}$O_2_ and mito$\dot{\text{D}}$O_2_ (Figure 2B). In detail, after recording mitoPO_2_ for about 20 s (one measurement per second), pressure was applied to the sensor to locally inhibit microcirculation. This resulted in a decrease of mitoPO_2_, which posed the basis for the assessment of mito$\dot{\text{V}}$O_2_. When mitoPO_2_ dropped near to zero or showed no further decline, pressure was maintained for a further 10 s. Thereafter, the pressure was released to allow local reoxygenation, which was followed by an increase of mitoPO_2_. This was used as the basis to obtain mito$\dot{\text{D}}$O_2_. The dynamic measurements were analyzed by two independent raters (PB, JH) with a self-developed program under MATLAB (MATLAB and Statistics Toolbox Release 2017a, The MathWorks, Inc. Natick, Massachusetts, United States). In short, the algorithm fits two complementary sigmoid functions, which model average mitoPO_2_ (without pressure), the decrease (during pressure) and the subsequent increase of mitoPO_2_ (after pressure release, see Figure 2B). Based on the fitted function parameters, average mito$\dot{\text{V}}$O_2_ and average mito$\dot{\text{D}}$O_2_ are derived as previously described (7). Pressure to the skin sensor during dynamic measurements was applied by a handheld dynamometer (Hoggan MicroFET2 Dynamometer, Hoggan Scientific LLc, Salt Lake City, United States) in order to achieve equal pressure in each dynamic measurement. Skin temperature was recorded as the temperature of the skin sensor.

**Figure 3:**
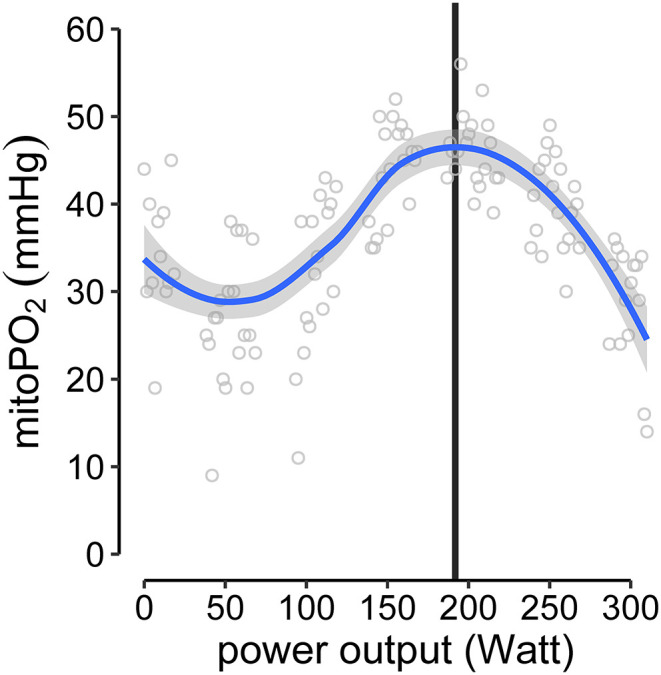
Example of MT-identification (mitochondrial threshold, vertical black line). Raw mitoPO_2_ values of single measurements were smoothed (locally estimated scatterplot smoothing, LOESS) and the maximum of the fitted curve was set as MT.

To analyze the individual kinetics of mitoPO_2_ from single measurements (every 10 s) during baseline and CPET, thereby excluding dynamic measurements, the data points were smoothed using locally estimated scatterplot smoothing (LOESS, span = 0.75, degree = 2). The mitochondrial threshold (MT) was set as maximum and/or decline of the fitted curve during CPET. An example is given in Figure 3 (Supplementary Figure 1 displays the data for all subjects).

#### Cardiopulmonary Exercise Test (CPET)

A bicycle ergometer was used (Custo-Cardio Diagnostik, Custo-Med GmbH, Ottobrunn, Germany). After calibration of the gas analyzer and flow sensor, baseline measurements were obtained, and the exercise test (graded multistage protocol) was started at 50 W with a workload increase of 50 W every 5 min. Participants were instructed to maintain a pedal frequency of 60–80 rotations per minute receiving feedback when being out of this scope. Exhaustion was deemed to have occurred when the participant could no longer maintain the required power output or wished to stop. Objective exhaustion levels and causes for the premature termination of the test were determined by classical criteria stated in various guidelines (25–27). Furthermore, the Borg Rating of Perceived Exertion (RPE) scale was used for the self-assessment of subjective exertion [from 6 (= no exertion at all) to 20 (= maximum exertion)] and dyspnea [from 6 (= no breathing difficulty) to 20 (= maximum breathing difficulty)] (28–30) at baseline and at the end of each load level. During the test, ventilation and standard gas exchange variables were recorded continuously on a breath-by-breath basis by a Ganshorn LF8 PowerCube® pneumotachograph (Ganshorn Medizin Electronic GmbH, Niederlauer, Germany) and displayed in the form of a 9-panel diagram according to Wassermann (31). The recorded and calculated variables were: minute ventilation ($\dot{\text{VE}}$), oxygen consumption ($\dot{\text{V}}$O_2_), carbon dioxide production ($\dot{\text{V}}$CO_2_), ventilatory equivalents $\dot{\text{V}}$E/$\dot{\text{V}}$O_2_ and $\dot{\text{V}}$E/$\dot{\text{V}}$CO_2_, partial pressure of end-tidal oxygen (PETO_2_) and carbon dioxide (PETCO_2_) and respiratory exchange ratio (RER). For data analysis the breath-by-breath data were exported as 10 s time-averaged values. Ventilatory thresholds (VT1 and VT2) were determined *post-hoc* using the method described by Westhoff et al. (17). For monitoring reasons according to guidelines (25, 26), a 12-lead ECG was recorded continuously throughout the test, blood pressure was measured non-invasively every 2.5 min and oxygen saturation was monitored continuously by pulse oximetry (S_p_O_2_).

#### Capillary Blood Gases (CBG)

After pre-treatment of the right earlobe with Finalgon® CPD Wärmecreme, 2 capillary blood samples (80 and 70 μl) for blood gas analysis were taken at baseline and after 3 min into each load level. One blood sample was analyzed on site using a GEM Premier 5000 blood gas testing system (Instrumentation Laboratory, Bedford, United States), the second blood sample was taken to a Radiometer ABL90FLEX blood gas analyzer (Radiometer GmbH, Krefeld, Germany) within the same building but about 75 s away from the study room. A third sample was used for a lactate measurement with a portable lactate meter (Lactate Scout 4, EKF-diagnostic GmbH, Barleben, Germany). Single measurements were obtained for base excess (BE), total concentration of hemoglobin (ctHb) and S_c_O_2_ (capillary oxygen saturation) (ABL90FLEX), double measurements for capillary partial pressure of oxygen and carbon dioxide (P_c_O_2_, P_c_CO_2_), pH (ABL90FLEX and GEM Premier 5000). Triple measurements were acquired for lactate (plus Lactate Scout 4) when possible.

Lactate thresholds (LT1 and LT2) were calculated from the capillary blood samples (measurements of the different samples were averaged) taken at each workload level. The evaluation of the lactate thresholds was done independently by the examiner (MH) using software written for performance diagnostics (Ergonizer, Vers. 5.7.3 Build 81, Freiburg, Germany). The Ergonizer software uses the model of Dickhuth, where the individual anaerobic threshold (IAS = LT2) is determined as a 1.5 mmol lactate increase above the minimal lactate equivalent (LT1) (32, 33).

### Statistical Analysis

In descriptive analysis, medians and first and third quartiles (Q_1_/Q_3_) are reported. For categorical variables we report absolute and relative frequencies.

To compare the variables of PpIX-TSLT, CPET (e.g., PETO_2_, PETCO_2_) and CBG between the different metabolic phases, we performed the following steps: The individual VT1 and VT2 were used to define the aerobic (all data points < VT1), the aerobic-anaerobic (all data points ≥VT1 and < VT2) and the anaerobic phase (all data points ≥VT2) for each subject. Due to the repeated measurements design and expected differences in baseline values, we used linear mixed models. In detail, the variables of PpIX-TSLT, CPET, and CBG served as dependent variables in the models. The phase was entered as categorical variable and served as independent variable (fixed effect). In addition, a random intercept for every subject was entered in the models. In the last step the estimated marginal means for the three phases were compared pairwise using the Tukey method.

To analyze the association between PpIX-TSLT variables and the variables of CPET and CBG we performed the following steps: All variables were standardized (mean ± SD: 0±1) before modeling (mixed models). In all models the PpIX-TSLT variables served as dependent variables. In the basic models the variables of CPET and CBG were entered as dependent variables (fixed effect). Here, the effect of the dependent variable was tested over all phases. In the next step, we obtained models estimating regression coefficients for every phase (phase specific models). Here the specific effect of the dependent variable was tested simultaneously for all phases. In the last step, the model fits of the basic models and the phase specific models were compared to test, if the phase specific models better explain the data. In addition, a random intercept for every subject was entered in all models. Because of the large number of tested regression coefficients, the *p*-values for mitoPO_2_, mito$\dot{\text{V}}$O_2_, and mito$\dot{\text{D}}$O_2_ were adjusted using the Bonferroni-Holm method.

The statistical analysis was performed with R [Version 3.5.1, Vienna, Austria (34)].

## Results

### Study Sample

Seventeen subjects participated in the study. Three subjects were excluded from the analysis for the following reasons: (a) wrong positioning of the ALA patch, which did not allow valid PpIX-TSLT measurements during CPET, (b) poor signal quality of PpIX-TSLT measurements for unknown reasons, (c) implausible CPET values during baseline measurements. The demographic characteristics of the 14 subjects in the final analysis sample are displayed in Table 1.

**Table 1:** Demographic characteristics of the study sample (median, Q_1|3_: first and third quartile) and frequencies of individual maximum power output levels during CPET.

**Variable**	**Median**	**Q_**1**_**	**Q_**3**_**	***n***
Age (years)	25.50	22.00	30.00	14
Weight (kg)	79.40	73.50	84.68	14
Height (m)	1.81	1.77	1.88	14
**Level**	**Maximum phase**		**Cumulative**	
	***n***	**%**	***n***	**%**
Baseline	0	0	14	100.0
50 (W)	0	0	14	100.0
100 (W)	0	0	14	100.0
150 (W)	0	0	14	100.0
200 (W)	4	28.6	14	100.0
250 (W)	5	35.7	10	71.4
300 (W)	5	35.7	5	35.7

### CPET-Derived Gas Exchange Measurements: Maximum Power Output Level, Ventilatory, and Metabolic Thresholds and Differences in PETO_2_ and PETCO_2_ Between the Different Metabolic Phases

Table 1 summarizes the maximum power output levels during CPET. Four subjects terminated CPET at 200 W and five subjects terminated CPET at 250 and 300 W, respectively. All subjects reached at least one of the objective termination criteria and the subjective termination criterion (Borg exertion, median: 18, Q_1|3_: 17|19).

Descriptive statistics on the obtained ventilatory and metabolic thresholds are displayed in Table 2. LT1 and VT1 were close to each other (median LT1: 116 W, median VT1: 111 W). LT2 was reached at a median of 156 W and VT2 was reached at a median of 238 W. The corresponding gas exchange variables at the thresholds are also depicted in Table 2. The individual VT1 and VT2 were used to define the individual aerobic (< VT1), aerobic-anaerobic (≥VT1 and < VT2), and anaerobic (≥VT2) phase.

**Table 2:** Descriptive statistics (median, Q_1|3_: first and third quartile) on ventilatory, metabolic, and mitochondrial thresholds.

**Variable**	**Statistic**	**Value**	**Power**	**Time**		**$\overset{.}{\text{V}}$O_**2**_**	**$\overset{.}{\text{V}}$CO_**2**_**	**RER**	**HR**	**RR**	**PETO_**2**_**	**PETCO_**2**_**
			**[W]**	**[s]**	**[%]**	**[l/min]**	**[l/min]**		**[/min]**	**[/min]**	**[mmHg]**	**[mmHg]**
LT1	Median	**2.0**	**116**	**406**	**40**	**1.13**	**1.07**	**0.91**	**110**	**20**	**96.35**	**42.05**
	Q_1_	1.2	95	274	35	0.87	0.78	0.88	94	17	95.00	41.09
	Q_3_	3.4	134	463	43	1.26	1.16	0.93	123	22	98.88	43.54
VT1	Median		**111**	**365**	**37**	**1.09**	**1.00**	**0.89**	**104**	**17**	**96.46**	**42.94**
	Q_1_		93	266	35	0.94	0.83	0.87	98	15	93.17	40.24
	Q_3_		118	428	40	1.22	1.10	0.92	117	19	98.61	44.74
LT2	Median	**3.5**	**156**	**639**	**56**	**1.40**	**1.37**	**0.99**	**136**	**19**	**98.66**	**43.50**
	Q_1_	2.7	140	538	52	1.25	1.27	0.95	120	17	97.04	42.06
	Q_3_	4.9	196	877	61	1.87	1.91	1.02	140	22	100.49	46.09
VT2	Median		**238**	**1140**	**83**	**2.16**	**2.26**	**1.06**	**162**	**23**	**100.30**	**46.94**
	Q_1_		214	999	82	2.05	2.17	1.02	154	22	97.09	44.08
	Q_3_		266	1290	88	2.41	2.65	1.10	174	25	101.35	48.46
MT	Median	**78**	**173**	**752**	**60**	**1.71**	**1.64**	**0.99**	**143**	**24**	**99.79**	**44.43**
	Q_1_	72	142	573	49	1.24	1.19	0.95	119	20	97.71	41.95
	Q_3_	83	190	857	70	1.83	1.95	1.07	154	26	100.66	45.38

*In addition, variables of gas exchange and physiological data at the corresponding threshold is shown*.

*LT1/2: first/second lactate threshold (value in mmol/l), VT1/2: first/second ventilatory threshold, MT: peak of mitochondrial oxygen tension (mitoPO_2_, value in mmHg), $\dot{\text{V}}$O_2_: oxygen uptake, $\dot{\text{V}}$CO_2_: carbon dioxide output, RER: respiratory exchange ratio, HR: heart rate, RR: respiratory rate, PETO_2_: end-tidal oxygen tension, PETCO_2_: end-tidal carbon dioxide tension. Median values are printed in bold*.

**Figure 4:**
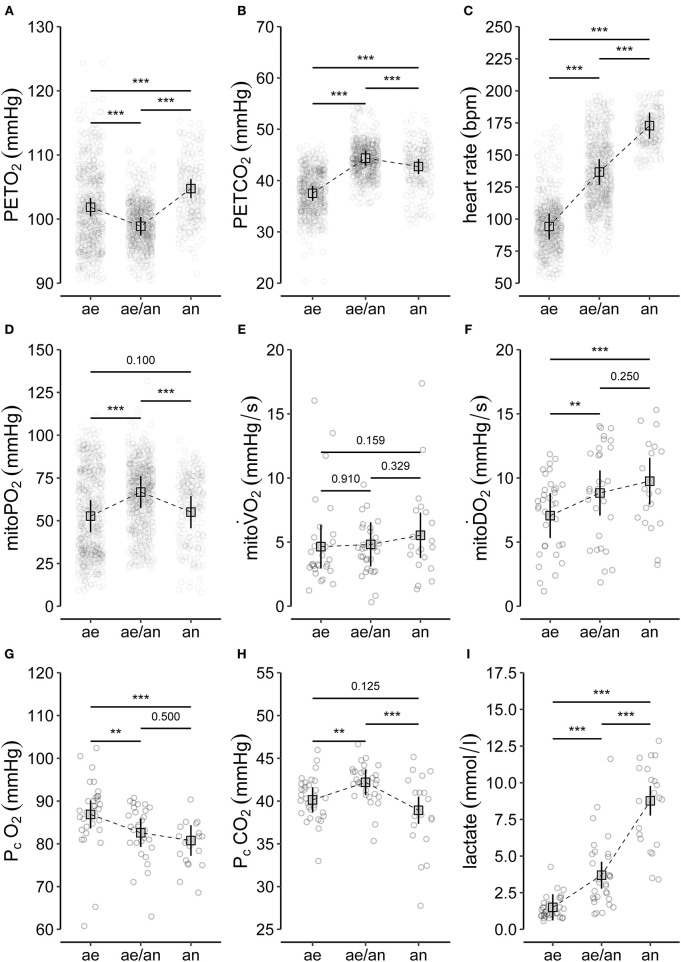
Summary of **(A–C)** selected variables of CPET (end-tidal oxygen tension, PETO_2_; end-tidal carbon dioxide tension, PETCO_2_; heart rate), **(D–F)** main variables of PpIX-TSLT measurements (mitochondrial oxygen tension, mitoPO_2_; mitochondrial oxygen consumption, mito$\dot{\text{V}}$O_2_; mitochondrial oxygen delivery, mito$\dot{\text{D}}$O_2_), and **(G–I)** variables of the capillary blood gas analysis (partial pressure of oxygen, P_c_O_2_; partial pressure of carbon dioxide, P_c_CO_2_; lactate). The dots refer to individual values of the variables during aerobic (ae, < VT1), aerobic-anaerobic (ae/an, ≥VT1 and < VT2), and anaerobic phase (an, ≥VT2). In addition, model predicted means (squares), corresponding 95% confidence intervals (lines) and adjusted *p*-values of comparisons between phases are displayed. *p < 0.05, **p < 0.01, ***p < 0.001.

Individual values and model predicted means of selected CPET variables for the different phases (aerobic, aerobic-anaerobic, anaerobic) are depicted in Figures 4A–C. There was a significant drop in mean PETO_2_ (Figure 4A) in the aerobic-anaerobic phase compared to the aerobic phase. This was followed by a significant increase in the anaerobic phase. Compared to the aerobic phase, mean PETO_2_ was significantly higher in the anaerobic phase.

Mean PETCO_2_ significantly increased in the aerobic-anaerobic phase compared to the aerobic phase (Figure 2B). This was followed by a significant decrease in the anaerobic phase. Compared to the aerobic phase, mean PETCO_2_ was significantly lower in the anaerobic phase.

With increasing power output, HR (Figure 4C) significantly increased from the aerobic via the aerobic-anaerobic to the anaerobic phase as physiologically expected. This was also true for $\dot{\text{V}}$O_2_, $\dot{\text{V}}$E, $\dot{\text{V}}$CO_2_, and RR. The detailed descriptive statistics of the main CPET variables are listed in Supplementary Table 1.

### PpIX-TSLT Measurements: Differences in MitoPO_2_, Mito$\dot{\text{V}}$O_2_, and Mito$\dot{\text{D}}$O_2_ Between the Metabolic Phases and Description of the Mitochondrial Threshold

Individual values and model predicted means of the main PpIX-TSLT variables for the different phases are presented in Figures 4D–F.

Mean mitoPO_2_ increased significantly from the aerobic to the aerobic-anaerobic phase (Figure 4D). This was followed by a significant decrease in the anaerobic phase, which resulted in non-significant mean differences between the aerobic and the anaerobic phase. Correspondingly, in 11 of 14 (78.6%) subjects, a peak followed by a final decrease or a decrease from a plateau (2/14, 14%) in mitoPO_2_ was identified (see Table 2 and Supplementary Figure 1). In analogy to ventilatory and metabolic thresholds, we termed this peak mitochondrial threshold (MT).

**Figure 5:**
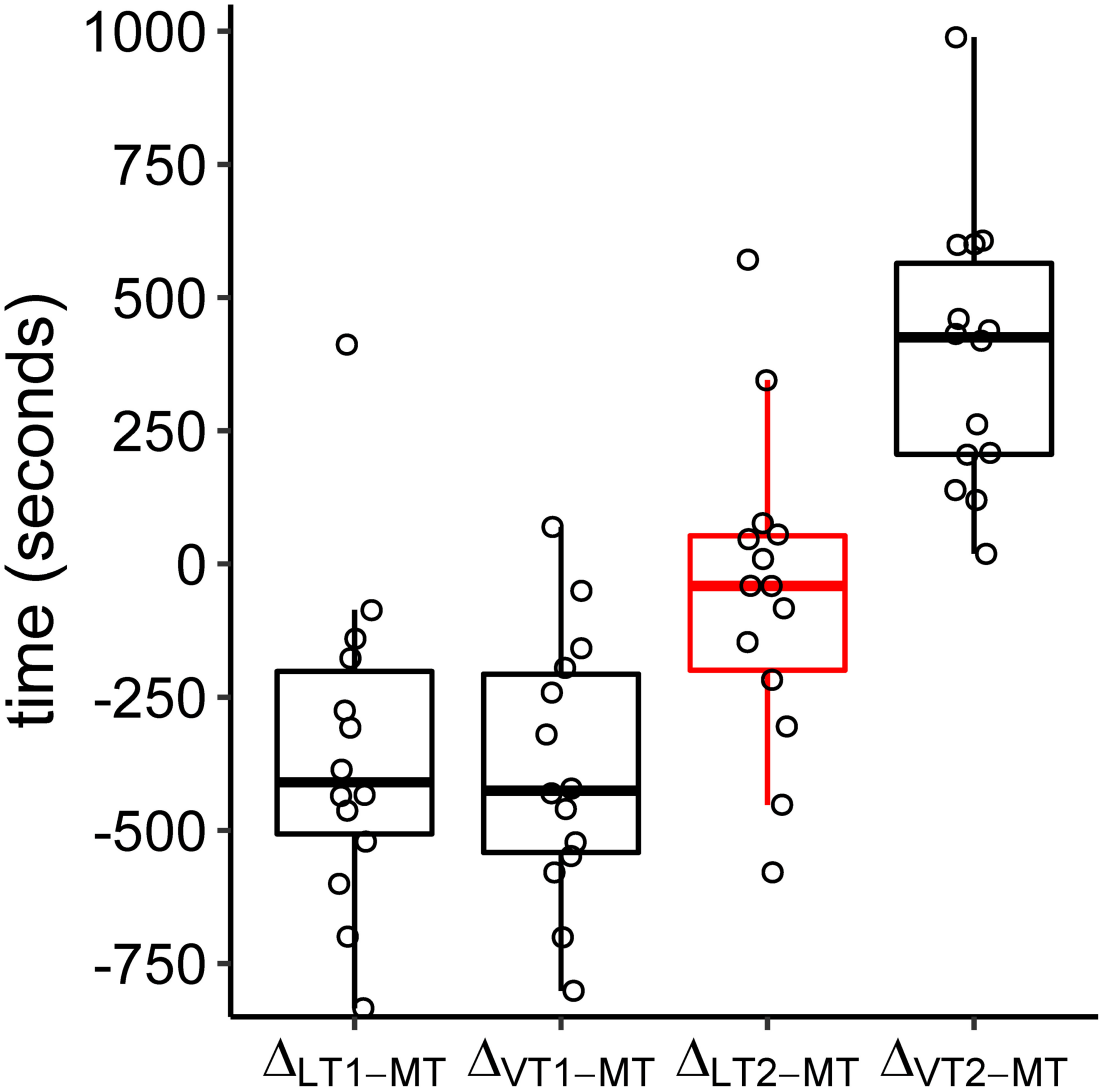
Boxplots of time differences in lactate (LT1, LT2), ventilatory (VT1, VT2), and mitochondrial (MT) thresholds. The circles refer to the individual differences. Negative values indicate a later occurrence of MT compared to the other thresholds.

**Figure 6:**
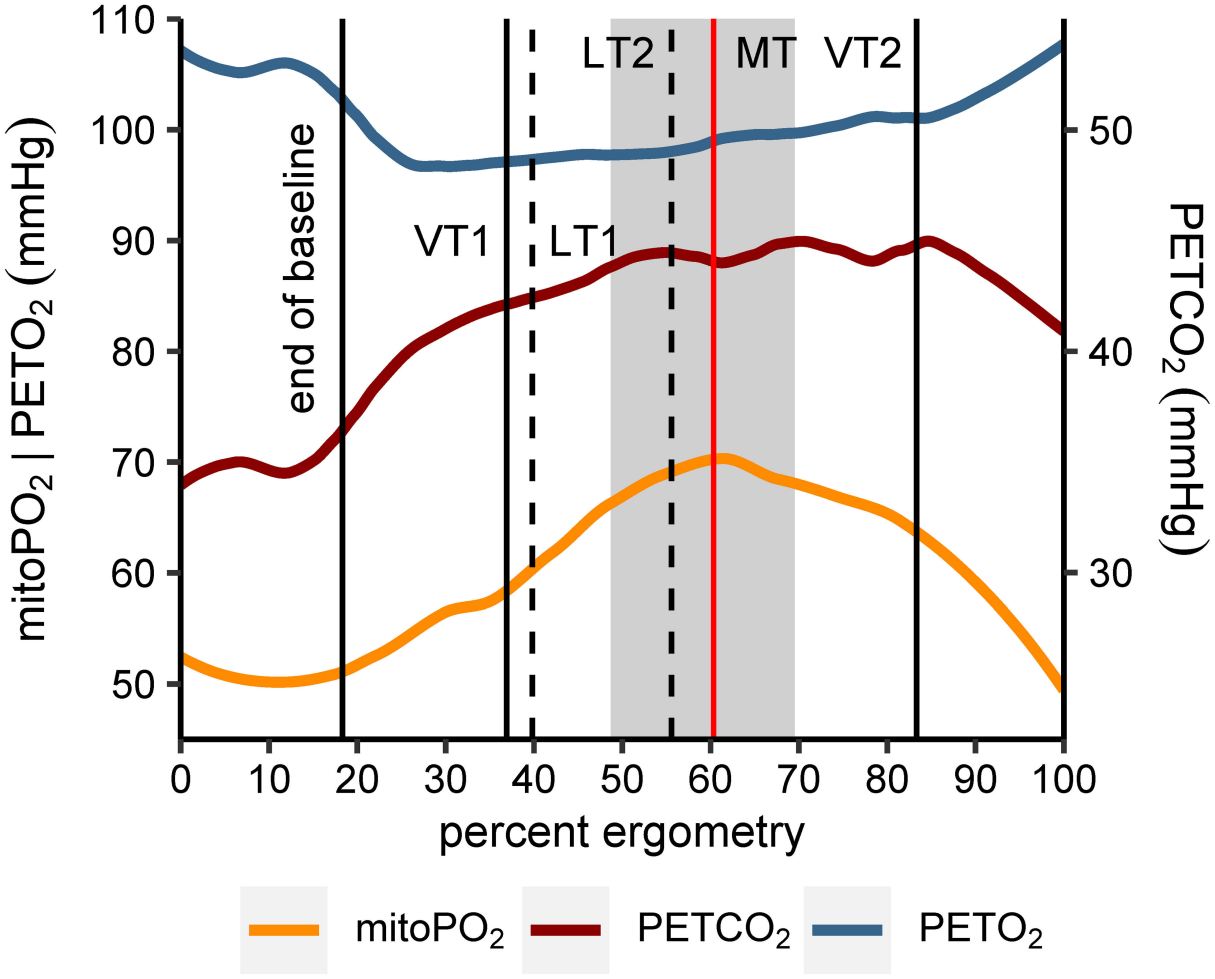
Smoothed course (locally estimated scatterplot smoothing, LOESS) of mitochondrial oxygen tension (mitoPO_2_), end-tidal oxygen tension (PETO_2_), and end-tidal carbon dioxide tension (PETCO_2_) for all subjects (*n* = 14). Values of the x-axis (percent ergometry) were scaled individually to the minimum (first measurement during baseline) and maximum power output level of the CPET (e.g., last measurement at 250 W). The vertical lines indicate end of baseline (median), first and second ventilatory and lactate threshold (medians of VT1|2, LT1|2) and mitochondrial threshold (median, MT). In addition, the gray area marks the interquartile range of MT.

The relative location of MT compared to ventilatory and metabolic thresholds is displayed in Figure 5. In 13 of the 14 subjects, MT occurred later than LT1 and VT1. In one participant, MT was recorded after LT1 but slightly before VT1. Median MT was located in the vicinity of LT2 (median difference in time: −42 s, Q_1|3_: −200 s | 53 s) and thus occurred slightly later but close to median LT2 at about 60% of maximum power output. In all subjects, MT occurred earlier than VT2. MitoPO_2_ dynamics, selected variables of gas exchange, and corresponding ventilatory thresholds during baseline and throughout load levels are summarized in Figure 6.

There were no significant differences in mean mito$\dot{\text{V}}$O_2_ between the three metabolic phases (Figure 4E).

Mean mito$\dot{\text{D}}$O_2_ increased significantly from the aerobic to the aerobic-anaerobic phase (Figure 4F). We found no significant differences in mean mito$\dot{\text{D}}$O_2_ values between the aerobic-anaerobic and the anaerobic phase, but mean mito$\dot{\text{D}}$O_2_ values were significantly higher in the anaerobic phase compared to the aerobic phase. The detailed descriptive statistics on the main variables of the PpIX-TSLT measurements are listed in Supplementary Table 2.

### Differences in P_c_O_2_, P_c_CO_2_, and Lactate Between the Metabolic Phases

Individual values and model predicted means of selected CBG variables for the different metabolic phases are presented in Figures 4G–I.

Compared to the aerobic phase, mean P_c_O_2_ was significantly lower in the aerobic-anaerobic and in the anaerobic phase (Figure 4G). There were no significant differences between the aerobic-anaerobic and the anaerobic phase for mean P_c_O_2_.

Mean P_c_CO_2_ was significantly higher in the aerobic-anaerobic phase compared to the aerobic phase (Figure 4H). This initial increase was followed by a significant decrease in the anaerobic phase. Mean P_c_CO_2_ in the anaerobic phase did not differ significantly between the aerobic and the anaerobic phase.

Mean lactate values increased significantly between the phases (Figure 4I). The detailed descriptive statistics on the main CBG variables are listed in Supplementary Table 3.

### Associations Between PpIX-TSLT-Derived Variables and Variables of CPET-Derived Gas Exchange and CBG

**Figure 7:**
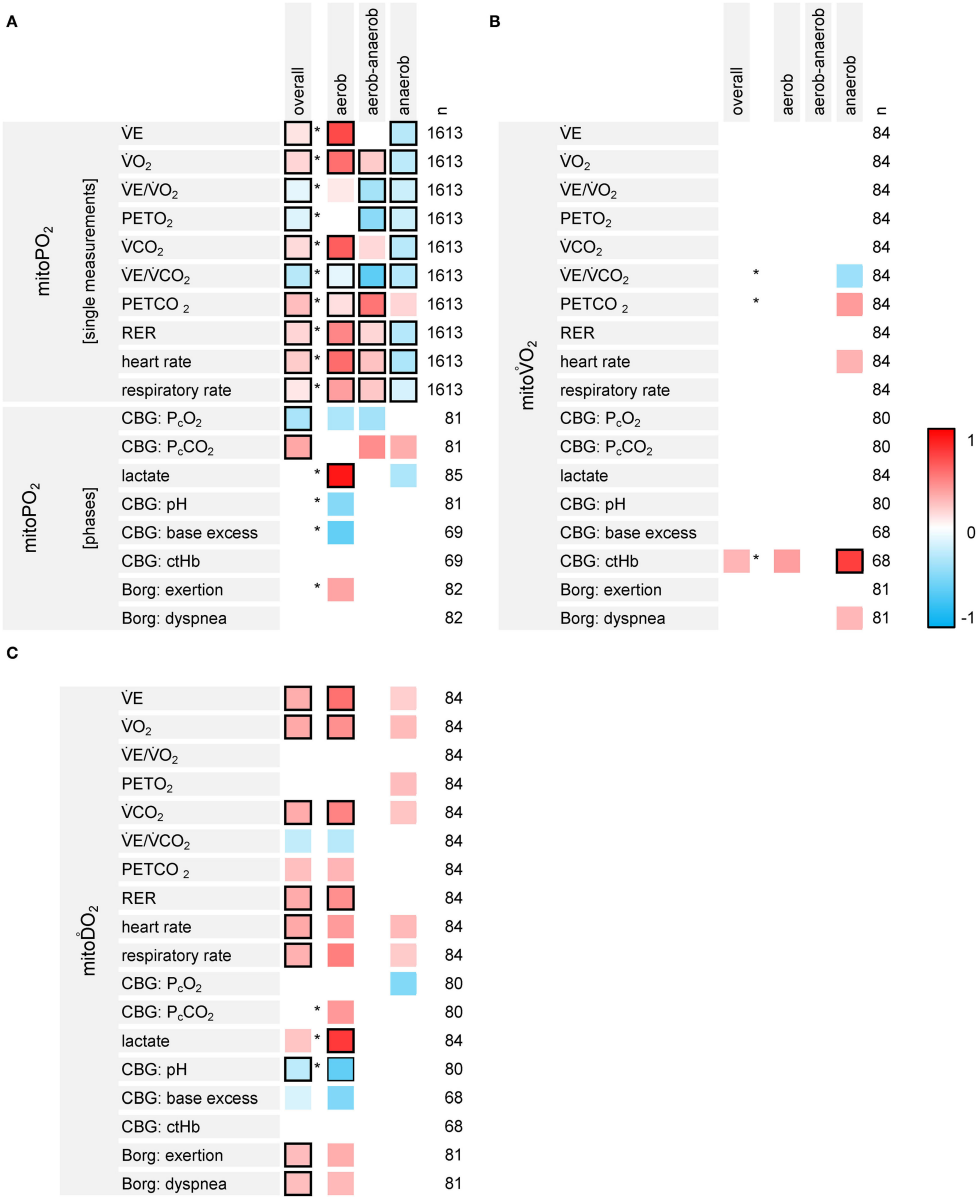
Results of the exploratory analyses on the associations between the variables of PpIX-TSLT measurements (**A**: mitoPO_2_, **B**: mito$\dot{\text{V}}$O_2_, **C**: mito$\dot{\text{D}}$O_2_), CPET-derived gas exchange and capillary blood gases (CBG). The first column lists the dependent variables, the second column displays the independent variables. The overall column indicates the standardized regression coefficients for the independent variable (one coefficient for all three phases, basic models). Asterisks indicate, if the models estimating separate regression coefficients for the phases show a better model fit compared to the basic models. The columns aerobic, aerobic-anaerobic and anaerobic display the corresponding standardized regression coefficients for each phase. The column n refers to the number (*n*) of data points, which were entered into the model. Regression coefficients with raw *p*-values ≥0.05 were set to zero (white). Framed cells indicate regression coefficients with Bonferroni-Holm adjusted *p*-values <0.05.

The results of the associative analyses are summarized in Figure 7.

MitoPO_2_ and CPET variables showed different associations throughout the different phases (detailed information is listed in Supplementary Table 4). CPET variables which increased over the different phases ($\dot{\text{V}}$E, $\dot{\text{V}}$O_2_, $\dot{\text{V}}$CO_2_, RER, RR, and HR) mainly were positively associated with mitoPO_2_ values within the aerobic and the aerobic-anaerobic phase. Most of these relations reversed within the anaerobic phase. $\dot{\text{V}}$E/$\dot{\text{V}}$CO_2_ and PETO_2_ were negatively associated with mitoPO_2_. PETCO_2_ was positively associated with mitoPO_2_. Nonetheless, there were differences in the strength of the associations between the phases (better model fit for the models with phase specific regression coefficients). P_c_CO_2_ was positively and P_c_O_2_ was negatively associated with mitoPO_2_. The models with specific effects for the phases did not show a better model fit. Lactate was positively associated with mitoPO_2_ values in the aerobic phase.

Mito$\dot{\text{V}}$O_2_ was negatively associated with $\dot{\text{V}}$E/$\dot{\text{V}}$CO_2_ and positively associated with PETCO_2_ in the anaerobic phase. We also found a positive association between mito$\dot{\text{V}}$O_2_ and ctHb. However, only the *p*-value for ctHb in the anaerobic phase survived adjustment for multiple testing.

Mito$\dot{\text{D}}$O_2_ was positively associated with variables showing an increase over the different phases ($\dot{\text{V}}$E, $\dot{\text{V}}$O_2_, $\dot{\text{V}}$CO_2_, RER, RR and HR, Borg scales). In addition, lactate showed a positive and pH showed a negative association with mito$\dot{\text{D}}$O_2_ in the aerobic phase.

## Discussion

In this study we performed PpIX-TSLT measurements simultaneously with a cardiopulmonary exercise test to analyze mitochondrial oxygen metabolism and its association with variables of gas exchange and CBG. We found significant differences in mitoPO_2_ and mito$\dot{\text{D}}$O_2_ between the three different phases during CPET. In the majority of subjects, we observed a peak in mitoPO_2_ which we termed mitochondrial threshold (MT). The peak occurred later than VT1 and LT1, but earlier than VT2. Its median appearance was timed around LT2. In the exploratory analysis we found significant associations between PpIX-TSLT variables and variables of CPET and CBG. Thus, our results clearly indicate an association between pulmonary respiration and cutaneous mitochondrial oxygen content during physical exercise.

### Kinetics of MitoPO_2_

In the group analysis, there was a significant increase of mitoPO_2_ between the aerobic and the aerobic-anaerobic phase, which was followed by a significant drop in mitoPO_2_ in the anaerobic phase. The increase of mitoPO_2_ during bicycle ergometry has already been described in our previous study (7), but a subsequent decline was not seen. This is possibly due to the submaximal intensity protocol instead of maximal physical exertion. Cutaneous mean mitoPO_2_ measurements at baseline (mean ± SD: 51.18 ± 21.93 mmHg) were slightly lower than previous values in humans acquired by the COMET system (7, 10). This might be due to differences in the study population [heterogeneous group pertaining to sex and age (7) and sepsis patients (10) vs. active young male participants] and the different measurement site (lumbar region vs. chest wall). The even lower values reported by Harms et al. (9) might be attributed to the use of a probe with a bigger measurement area in our study [COMET device (photonicshealthcare.com) vs. a clinical prototype device]. However, the variation of baseline mitoPO_2_ was similar.

### Mitochondrial (MT), Metabolic (LT1 and LT2), and Ventilatory (VT1 and VT2) Thresholds

In the majority of the participants, the mitochondrial oxygen tension increased during exercise until a peak (MT) was reached and then dropped again. In sports medicine, lactate thresholds are used to determine training intensities for endurance training assuming that LT2 denotes more or less accurately the maximum lactate steady state at which lactate production and elimination are still balanced. In the review of Faude et al. (18), it is speculated that working intensities near LT2 may induce a considerable increase in the oxidative metabolism of muscle cells, although anaerobic glycolysis is enhanced. Prolonged training at this power output intensity might stimulate aerobic metabolism in muscle cells and thus pose an appropriate level for endurance training. However, there is still debate on the correct determination of LT2 (18). A relatively new concept in training prescription is the identification and determination of training intensities near the critical power (CP), the highest power output which can be sustained for a longer period of time under predominantly aerobic conditions (35). In a most recent meta-analysis, the point in time of reaching CP was located between the maximal lactate steady state and RCP/VT2 (36), which would be in line with the temporal occurrence of MT in our study. The idea of using the moment of decline in mitochondrial performance might pose a new tool of validating LT2 or even the moment, when critical power is reached. To our knowledge, the concept of a mitochondrial threshold has not been proposed before. However, in the study of Römers et al. mitoPO_2_ in hemodiluted pigs also behaved threshold-like (37). Further studies are needed to evaluate these findings.

### MitoPO_2_ and Its Relation to CPET-Derived Gas Exchange Variables

To our knowledge, data on associations of mitochondrial oxygen tension with CPET-derived gas exchange parameters hitherto do not exist. We were able to demonstrate a phase-dependent association of mitoPO_2_ with $\dot{\text{V}}$O_2_ and $\dot{\text{V}}$CO_2_. During the aerobic phase, these parameters were positively associated, reflecting the balance between oxygen uptake and carbon dioxide elimination on the one side and the simultaneous increase of mitochondrial oxygen content on the other side. During the aerobic-anaerobic transition phase, the positive correlation of $\dot{\text{V}}$O_2_ with mitoPO_2_ continued while a correlation with $\dot{\text{V}}$CO_2_ was no longer statistically significant after *p*-value adjustment. This might indicate the increasing accumulation and expiration of additional CO_2_ accruing from the beginning anaerobic glycolysis. During the anaerobic phase, both parameters were negatively associated with mitoPO_2_. With rising levels of lactic acid, a huge amount of CO_2_ is produced by the buffering of H^+^ ions, so that the intensity of respiration is mainly driven by the need to eliminate the excess CO_2_ (14). At the same time, mitoPO_2_–derived from mitochondria in skin—declined despite a further increase in $\dot{\text{V}}$O_2_. One reason could be that during anaerobic metabolism mitochondrial oxygen demand is no longer matched by supply. Another possible explanation might be that under the conditions of extreme physical exertion, mainly muscular mitochondria are in need of oxygen and therefore, a shift of the scarce substrate from skin to muscles is initiated. According to Boushel et al. muscular mitochondrial respiratory capacity exceeds maximal oxygen delivery in humans (38), thus resulting in a competitive situation between muscles and less active organs. Golub et al. proposed that oxygen demand increases oxygen supply by incremented diffusion gradients and a compensatory vascular response (39), which might support the above-mentioned hypothesis. The simultaneous mitoPO_2_ measurement in muscular and cutaneous mitochondria might provide clarification. The closest positive association was seen between mitoPO_2_ and PETCO_2_ for all phases, the best negative association was between mitoPO_2_ and $\dot{\text{V}}$E/$\dot{\text{V}}$CO_2_ followed by mitoPO_2_ and PETO_2_. The latter could be accustomed for by the mitochondrial oxygen consumption, which consequently leads to a lower oxygen content in the exhaled air. Since PETCO_2_ behaves contrary to PETO_2_, this possibly explains the association without being causative.

### MitoPO_2_ and Its Relation to CBG Variables

MitoPO_2_ was negatively associated with P_c_O_2_ in the aerobic and aerobic-anaerobic phases. Despite a decrease of the mean P_c_O_2_ over the three phases, mean mitoPO_2_ at first increased from the aerobic to the aerobic-anaerobic phase and then decreased again in the anaerobic phase. In a hemodilution study in pigs, Römers et al. found a sudden drop in mitoPO_2_ at a critical stage of hemodilution. Although Hb declined with each hemodilution step, a mitochondrial response was only seen at that critical point in time (37), hinting at a certain independence of mitochondrial oxygen tension from Hb levels and thus from arterial oxygen content before this moment. In contrast, P_c_CO_2_ was positively associated throughout all phases. Since P_a_CO_2_ is more or less consistent with P_A_CO_2_ (alveolar carbon dioxide partial pressure), which also is the same as PETCO_2_ in healthy persons (14), this was expected. Only during the aerobic phase, lactate was positively associated with mitoPO_2_. During aerobic-anaerobic transition, when anaerobic glycolysis starts and lactate levels rise, lactate was no longer associated with mitoPO_2_. An even negative association was found during the anaerobic phase due to increasing lactate levels whilst mitoPO_2_ decreased.

### Mito$\dot{\text{V}}$o_2_: Kinetics and Relation to Variables of CPET-Derived Gas Exchange and CBG

We observed no significant changes in mito$\dot{\text{V}}$O_2_ during exercise. In the study of Baumbach et al. mito$\dot{\text{V}}$O_2_ had significantly decreased in post-exercise measurements compared to baseline (7). However, no measurements were obtained during exercise. The study also differed insofar that only a submaximal power output was generated and that multiple measurements were done at each time point. Mito$\dot{\text{V}}$O_2_ measurements at baseline (4.88 ± 3.00 mmHg) were in the range of previous measurements (7, 9–11). Of the CBG parameters only ctHb showed a positive association, indicating that mito$\dot{\text{V}}$O_2_ might depend on the oxygen transport capacity of the blood and thus the overall availability of oxygen.

### MitoḊO_2_: Kinetics and Relation to Variables of CPET-Derived Gas Exchange and CBG

Mito$\dot{\text{D}}$O_2_ increased from baseline to maximum exertion. In the study of Baumbach et al. mito$\dot{\text{D}}$O_2_ tended to increase from baseline to post-exercise phases, but the increase was not significant (7). Again, the submaximal power output in that study and differences in the measurement protocol might explain the differences. Mito$\dot{\text{D}}$O_2_ values at baseline were 6.78 ± 3.2 and comparable to results from Baumbach et al. (7) and Neu et al. (10). Since the cardiovascular system is responsible for the increase in oxygen delivery to the tissues (40), and since mito$\dot{\text{D}}$O_2_ mainly reflects reperfusion and thus reoxygenation after temporary interruption of microcirculation, its positive association with HR was expected. A positive association with $\dot{\text{V}}$E, $\dot{\text{V}}$O_2_ and $\dot{\text{V}}$CO_2_ also was logical, because intensified ventilation is followed by increased O_2_-uptake and also increased CO_2_-elimination. However, the continuing increase of mito$\dot{\text{D}}$O_2_, while mitoPO_2_ decreased, might be indicative of a true limit of the system. Despite an accelerated oxygen delivery, the demand cannot be met. Mito$\dot{\text{D}}$O_2_ increase might be attributed to a facilitated oxygen release when pH decreases and body temperature rises (41) as can be observed during high intensity exercise. In order to dispense of the extra heat generated by the physical exercise, skin perfusion is enhanced by vasodilation of cutaneous vessels and inhibition of the vasoconstrictor tone (40). This also might contribute to a rise in the slope of cutaneous mitoPO_2_ increase after pressure release.

### Limitations and Perspective

In the set-up of our trial, mitoPO_2_ and its derivatives were analyzed in skin, not muscle. Research by Mellstrom et al. (42) and Venkatesh et al. (43) demonstrated that subcutaneous tissue PO_2_ compares to that of intestinal mucosa and to ileal luminal PO_2_, respectively. The rationale would be that oxygen tension in skin can be used as an indicator of the conditions in other organs. However, according to clinical and pre-clinical studies in oncologic dermatology, 5-ALA can only penetrate the epidermis and the upper layers of the dermis (44), making it difficult to translate the results of previous investigations to our findings. In addition, PpIX-TSLT measurements with the COMET device are restricted to a depth of about 0.1 mm in the epidermis (8). In a recent study in rats, Wefers-Bettink et al. (45) found similar mitoPO_2_ values measured with the probe placed directly on muscle compared to simultaneous measurements in abdominal skin at baseline. Yet, after 3 h, muscle mitoPO_2_ was markedly higher than skin mitoPO_2_. This was attributed to wound healing, nonetheless indicating that mitochondria in different organs can behave differently depending on local conditions. However, physical exercise is not limited to muscles but is a condition that affects the whole organism. Further studies will be needed to determine, if measurements of mitoPO_2_ in epidermal cells represent an overall metabolic marker in the sense of a training relevant or disease severity indicating threshold.

Hitherto, only little evidence is available regarding intra- and interindividual variance in the absolute mitoPO_2_ values (7, 9). The same is true for the magnitude of mitoPO_2_ measured by PpIX-TSLT: earlier methodically different measurements and estimates ranged lower by one order of magnitude (46, 47). We found a large interindividual variance in COMET-derived mitoPO_2_ measurements, which also was reported in previous studies (7, 10). The COMET system is a novel device for which influencing factors are studied only to some extent and in small groups of participants. The measurements deviate from previous measurements with a prototype device (9). Moreover, normal values do hitherto not exist. Therefore, further studies with larger cohorts of healthy people are required to define reference values and influencing variables. Overall, mean changes in mitoPO_2_ and mito$\dot{\text{D}}$O_2_ during the phases were highly significant, but compared to previously published data on stability of these variables (mitoPO_2_ | mito$\dot{\text{D}}$O_2_, standard error of measurement: 10 mmHg | 1.5 mmHg/s, minimum detectable difference: 30 mmHg | 4 mmHg/s) the differences were rather small. In accordance to the high interindividual variance, we also observed a high intraindividual variance regarding changes in mitoPO_2_ over the metabolic phases (Supplementary Figure 1).

We found several statistically significant associations between variables of PpIX-TSLT, gas exchange and CBG. Nonetheless, only a small proportion of the variance (48) in mitoPO_2_, mito$\dot{\text{D}}$O_2_ and mito$\dot{\text{V}}$O_2_ could be explained by the independent variables (${\text{R}}_{\text{marginal}}^{2}$ for all models <0.15, median: 0.07, Q_1|3_: 0.046 | 0.104). As described in the last paragraph, interindividual differences (modeled as random intercept) explained a much larger part of the variance (${\text{R}}_{\text{conditional}-\text{marginal}}^{2}$, median: 0.56, Q_1|3_: 0.46 | 0.61).

In our study we chose a graded exercise protocol with a relatively long duration of each load level in order to achieve a steady state. It is known that this type of protocol is favorable for blood lactate determination (49) but no so valuable for the identification of ventilatory thresholds (50). Nonetheless, it was shown in several studies that the determination of ventilatory thresholds is also feasible, when an incremental test design with steps of longer duration is used (32, 51).

Although a mitochondrial threshold was identified in the majority of subjects, its exact timing depended on the smoothing algorithm and might have been influenced by outlier values. Furthermore, mitoPO_2_ single measurements might have been influenced to some degree by the intermediate dynamic measurements (e.g., rebound effect after reoxygenation). The measurements should be repeated using different protocols, e.g., ramp vs. stepwise protocol or shortened duration of workload levels, in order to create a more continuous rise in power output intensity. Furthermore, mitoPO_2_ single measurements should not be interrupted by dynamic measurements. The simultaneous use of a second COMET system might be a reasonable alternative.

In our study population $\dot{\text{V}}$O_2max_ on average was lower than mostly reported in the literature (see Supplementary Table 5). E.g., according to Heyward, a relative $\dot{\text{V}}$O_2max_ of 42–45 ml/kg/min in 20 to 29-year-old men compares to a just moderate fitness level (52). In line with this, physically active students reached a $\dot{\text{V}}$O_2max_ of about 49 ml/kg/min in a study of Boone et al. (53). In contrast, Zoll et al. reported a $\dot{\text{V}}$O_2max_ of 36 ml/kg/min in physically active volunteers (54), which is more congruent with our findings. According to the recently in Germany executed population based Study of Health in Pomerania (SHIP) (55), which sought to implement reference values for CPET, the $\dot{\text{V}}$O_2max_ values of the participants in our study were above the 5th quantile of age-, sex-, and weight-adjusted expected values. The following mechanisms and conditions might have affected $\dot{\text{V}}$O_2max_: We did not ask the participants to refrain from exercise for 48 h prior to the test, nor did we give dietary instructions. The time of day varied among the tests. All three aspects are known to influence gas exchange parameters (14, 56). In addition, the relatively long duration of the test and also of the single load increments as well as the type of protocol (graded increments vs. ramp) might have contributed to our diverging results (53, 56, 57). Another mechanism that might have had an impact on $\dot{\text{V}}$O_2max_ was the presence of light to moderate exercise induced arterial hypoxemia (EIAH) in 6 participants assessed by S_c_O_2_. This condition is found mainly in habitually active persons but also in athletes at a percentage of up to 50% and can negatively influence $\dot{\text{V}}$O_2max_ (58). However, our study set-up was designed as a pilot study to test gas exchange and metabolic variables against a novel technique for measuring mitoPO_2_. Thus, the absolute values of the mentioned variables were of minor importance.

Last but not least, the question remains how our findings can be put into the context of intensive care medicine and serious systemic disease such as sepsis. The mitochondrial threshold possibly indicates a breakpoint in mitochondrial performance. As such, the MT might well be a marker for the point of no return in the course of disease. It has already been shown that in planned procedures like, e.g., surgery, monitoring of mitoPO_2_ is feasible and more sensitive than classical parameters (8). In the case of sepsis however, this would require the possibility of early and continuous monitoring. This would necessitate a faster uptake of 5-ALA in cutaneous cells and a prolonged availability after a one-time-application. Pharmacologic modifications to the 5-ALA patch could solve the problem. If it is possible to overcome these difficulties, the technique might develop into a useful instrument for the timing of interventions in critical illness.

## Conclusion

In this pilot study we demonstrated that PpIX-TSLT measurements of mitoPO_2_, mito$\dot{\text{V}}$O_2_, and mito$\dot{\text{D}}$O_2_ with the COMET device are feasible simultaneously with CPET. Furthermore, we showed that mitoPO_2_ and mito$\dot{\text{D}}$O_2_ measured in skin are associated with gas exchange and CBG derived variables during exercise to exhaustion. Interestingly, we found a decline in mitoPO_2_ after a peak or plateau (MT), which occurred between VT1 and VT2 around LT2. Because aerobic energy production originates from mitochondria, this phenomenon might well be indicative of a breakpoint in performance and could pose a valuable instrument in performance diagnostics as well as in serious disease. As our study population comprised of only 14 subjects, the results will have to be validated in larger cohorts and future studies should aim to relate mitochondrial oxygen kinetics not only to parameters of high-performance sports but also to such of critical illness.

## Data Availability Statement

The raw data supporting the conclusions of this article will be made available by the authors, without undue reservation.

## Ethics Statement

The study involving human participants was reviewed and approved by the Ethics Committee of the Friedrich Schiller University Jena (2019-1296-BO). The participants provided their written informed consent to participate in this study.

## Author Contributions

CS-W, PB, MH, and SC: conception and design of the study. JH, PB, and SD: organization and performance of measurements. PB, CS-W, JH, and MH: data analysis. PB: statistical analysis. CS-W, PB, and SC: drafting the manuscript for important intellectual content. PB, CS-W, JH, SD, NB, MH, and SC: revising the manuscript prior to submission. All authors carefully reviewed and approved the manuscript.

## Conflict of Interest

The authors declare that the research was conducted in the absence of any commercial or financial relationships that could be construed as a potential conflict of interest.

## Supplementary Material

Supplementary Figure 1 Smoothed course of mitochondrial oxygen partial pressure (mitoPO_2_) during cardiopulmonary exercise testing for every study subject. The gray dots indicate raw mitoPO_2_ values and the blue line indicates the fitted regression line using locally estimated scatterplot smoothing (LOESS, span = 0.75, degree = 2). The gray area depicts the 95% confidence interval. The individual mitochondrial threshold is marked with the black vertical line. It marks the maximum (ID 1, 2, 5, 6, 8, 9, 10, 11, 12, 15, 16) or local maximum (ID 3, 4, 13) of the fitted regression line.

Supplementary Tables 1–3 Detailed descriptive statistics on all variables of CPET, PpIX-TSLT measurements and capillary blood gas analyses and estimated marginal means and contrasts for selected variables of CPET, PpIX-TSLT measurements and blood gas analyses.

Supplementary Table 4 Coefficients of the regression models analyzing the association of variables of PpIX-TSLT measurement, CPET and capillary blood gas analyses.

Supplementary Table 5 Detailed description of the V°O_2max_ for all subjects.

## References

[1] Singer M. The role of mitochondrial dysfunction in sepsis-induced multi-organ failure. Virulence. (2014) 5:66–72. 10.4161/viru.26907 24185508 PMC3916385

[2] Stanzani G Duchen MR Singer M. The role of mitochondria in sepsis-induced cardiomyopathy. Biochim Biophys Acta Mol Basis Dis. (2019) 1865:759–73. 10.1016/j.bbadis.2018.10.011 30342158

[3] Zhang H Feng YW Yao YM. Potential therapy strategy: targeting mitochondrial dysfunction in sepsis. Mil Med Res. (2018) 5:41. 10.1186/s40779-018-0187-0 30474573 PMC6260865

[4] Granata C Jamnick NA Bishop DJ. Training-induced changes in mitochondrial content and respiratory function in human skeletal muscle. Sports Med. (2018) 48:1809–28. 10.1007/s40279-018-0936-y 29934848

[5] Mik EG Stap J Sinaasappel M Beek JF Aten JA van Leeuwen TG. Mitochondrial PO_2_ measured by delayed fluorescence of endogenous protoporphyrin IX. Nat Methods. (2006) 3:939–45. 10.1038/nmeth940 17060918

[6] Harms FA Bodmer SI Raat NJ Mik EG. Non-invasive monitoring of mitochondrial oxygenation and respiration in critical illness using a novel technique. Crit Care. (2015) 19:343. 10.1186/s13054-015-1056-9 26391983 PMC4578612

[7] Baumbach P Neu C Derlien S Bauer M Nisser M Buder A. A pilot study of exercise-induced changes in mitochondrial oxygen metabolism measured by a cellular oxygen metabolism monitor (PICOMET). Biochim Biophys Acta Mol Basis Dis. (2019) 1865:749–58. 10.1016/j.bbadis.2018.12.003 30593898

[8] Ubbink R Bettink MAW Janse R Harms FA Johannes T Munker FM. A monitor for Cellular Oxygen METabolism (COMET): monitoring tissue oxygenation at the mitochondrial level. J Clin Monit Comput. (2017) 31:1143–50. 10.1007/s10877-016-9966-x 28000040 PMC5655595

[9] Harms FA Stolker RJ Mik EG. Cutaneous respirometry as novel technique to monitor mitochondrial function: a feasibility study in healthy volunteers. PLoS ONE. (2016) 11:e0159544. 10.1371/journal.pone.0159544 27455073 PMC4959702

[10] Neu C Baumbach P Plooij AK Skitek K Götze J von Loeffelholz C. Non-invasive assessment of mitochondrial oxygen metabolism in the critically ill patient using the protoporphyrin IX-triplet state lifetime technique—a feasibility study. Front Immunol. (2020) 11:757. 10.3389/fimmu.2020.00757 32457741 PMC7221153

[11] van Diemen MPJ Berends CL Akram N Wezel J Teeuwisse WM Mik BG. Validation of a pharmacological model for mitochondrial dysfunction in healthy subjects using simvastatin: A randomized placebo-controlled proof-of-pharmacology study. Eur J Pharmacol. (2017) 815:290–7. 10.1016/j.ejphar.2017.09.031 28943100

[12] van Dijk LJD Ubbink R Terlouw LG van Noord D Mik EG Bruno MJ. Oxygen-dependent delayed fluorescence of protoporphyrin IX measured in the stomach and duodenum during upper gastrointestinal endoscopy. J Biophotonics. (2019) 12:e201900025. 10.1002/jbio.201900025 31140739 PMC7065646

[13] Alveolar gas equation. In: Huang C Chambers D Matthews G, (editors). Basic Physiology for Anaesthetists. 2nd ed Cambridge: Cambridge University Press (2019). p. 77–9.

[14] Kroidl RF Schwarz S Lehnigk BJF (editors). Kursbuch Spiroergometrie, Technik und Befundung verständlich gemacht Stuttgart: Thieme Verlag (2015).

[15] Antonutto G Di Prampero PE. The concept of lactate threshold. A short review. J Sports Med Phys Fitness. (1995) 35:6–12. 7474995

[16] Skinner JS McLellan TH. The transition from aerobic to anaerobic metabolism. Res Q Exerc Sport. (1980) 51:234–48. 10.1080/02701367.1980.10609285 7394286

[17] Westhoff M Ruhle KH Greiwing A Schomaker R Eschenbacher H Siepmann M. Positional paper of the German working group “cardiopulmonary exercise testing” to ventilatory and metabolic (lactate) thresholds. Dtsch Med Wochenschr. (2013) 138:275–80. 10.1055/s-0032-1332843 23361352

[18] Faude O Kindermann W Meyer T. Lactate threshold concepts: how valid are they? Sports Med. (2009) 39:469–90. 10.2165/00007256-200939060-00003 19453206

[19] Thomas S Reading J Shephard RJ. Revision of the Physical Activity Readiness Questionnaire (PAR-Q). Can J Sport Sci. (1992) 17:338–45. 1330274

[20] Mik EG. Special article: measuring mitochondrial oxygen tension: from basic principles to application in humans. Anesth Analg. (2013) 117:834–46. 10.1213/ANE.0b013e31828f29da 23592604

[21] Stern O Volmer M Über die Abklingungszeit der Fluoreszenz. Phys Z. (1919) 20:183–8.

[22] Poulson R. The enzymic conversion of protoporphyrinogen IX to protoporphyrin IX in mammalian mitochondria. J Biol Chem. (1976) 251:3730–3. 6461

[23] Wachowska M Muchowicz A Firczuk M Gabrysiak M Winiarska M Wanczyk M Aminolevulinic acid (ALA) as a prodrug in photodynamic therapy of cancer. Molecules. (2011) 16:4140–64. 10.3390/molecules16054140

[24] Donnelly RF McCarron PA Woolfson AD. Drug delivery of aminolevulinic acid from topical formulations intended for photodynamic therapy. Photochem Photobiol. (2005) 81:750–67. 10.1562/2004-08-23-IR-283R1.1 15790300

[25] American College of Sports Medicine Guidelines for Exercise Testing and Prescription. Philadelphia, PA: Lea & Febiger (1986).

[26] Fletcher GF Ades PA Kligfield P Arena R Balady GJ Bittner VA. Exercise standards for testing and training: a scientific statement from the American Heart Association. Circulation. (2013) 128:873–934. 10.1161/CIR.0b013e31829b5b44 23877260

[27] Klingenheben T Löllgen H Bosch R Trappe HJ Manual zum Stellenwert der Ergometrie. Der Kardiol. (2018) 12:342–55. 10.1007/s12181-018-0265-2

[28] Borg G Anstrengungsempfinden und körperliche Aktivität. Dtsch Arztebl Int. (2004) 101:A-1016.

[29] Borg GA. Psychophysical bases of perceived exertion. Med Sci Sports Exerc. (1982) 14:377–81. 10.1249/00005768-198205000-00012 7154893

[30] Löllgen H Fahrenkrog I Löllgen D Bewertung ergometrischer Größen. In: Löllgen H Erdmann E Gitt AK, editors. Ergometrie. 3. Heidelberg: Springer Medizin Verlag (2010). p. 71 10.1007/978-3-540-92730-3

[31] Binder RK Wonisch M Corra U Cohen-Solal A Vanhees L Saner H. Methodological approach to the first and second lactate threshold in incremental cardiopulmonary exercise testing. Eur J Cardiovasc Prev Rehabil. (2008) 15:726–34. 10.1097/HJR.0b013e328304fed4 19050438

[32] Dickhuth HH Yin L Niess A Röcker K Mayer F Heitkamp HC. Ventilatory, lactate-derived and catecholamine thresholds during incremental treadmill running: relationship and reproducibility. Int J Sports Med. (1999) 20:122–7. 10.1055/s-2007-971105 10190774

[33] Dickhuth H-H Huonker M Münzel T Drexler H Berg A Keul J Individual anaerobic threshold for evaluation of competitive athletes and patients with left ventricular dysfunction. In: Bachl N Graham TE Löllgen H, editors. Advances in Ergometry. Berlin; Heidelberg; New York, NY: Springer (1991). p. 173–9. 10.1007/978-3-642-76442-4_26

[34] R Development Core Team R: A Language and Environment for Statistical Computing. Vienna: R Foundation for Statistical Computing (2013).

[35] Poole DC Burnley M Vanhatalo A Rossiter HB Jones AM. Critical power: an important fatigue threshold in exercise physiology. Med Sci Sports Exerc. (2016) 48:2320–34. 10.1249/MSS.0000000000000939 27031742 PMC5070974

[36] Galán-Rioja M González-Mohíno F Poole DC González-Ravé JM. Relative proximity of critical power and metabolic/ventilatory thresholds: systematic review and meta-analysis. Sports Med. (2020) 50:1771–83. 10.1007/s40279-020-01314-8 32613479

[37] Romers LH Bakker C Dollee N Hoeks SE Lima A Raat NJ. Cutaneous mitochondrial PO_2_, but not tissue oxygen saturation, is an early indicator of the physiologic limit of hemodilution in the pig. Anesthesiology. (2016) 125:124–32. 10.1097/ALN.0000000000001156 27176212

[38] Boushel R Gnaiger E Calbet JA Gonzalez-Alonso J Wright-Paradis C Sondergaard H. Muscle mitochondrial capacity exceeds maximal oxygen delivery in humans. Mitochondrion. (2011) 11:303–7. 10.1016/j.mito.2010.12.006 21147270

[39] Golub AS Tevald MA Pittman RN. Phosphorescence quenching microrespirometry of skeletal muscle *in situ*. Am J Physiol Heart Circ Physiol. (2011) 300:H135–43. 10.1152/ajpheart.00626.2010 20971766 PMC3023268

[40] Burton DA Stokes K Hall GM Physiological effects of exercise. Contin Educ Anaesth Crit Care Pain. (2004) 4:185–8. 10.1093/bjaceaccp/mkh050

[41] Forbes WH Roughton FJ. The equilibrium between oxygen and haemoglobin: I. The oxygen dissociation curve of dilute blood solutions. J Physiol. (1931) 71:229–60. 10.1113/jphysiol.1931.sp002729 16994173 PMC1403062

[42] Mellstrom A Månsson P Jonsson K Hartmann M. Measurements of subcutaneous tissue PO_2_ reflect oxygen metabolism of the small intestinal mucosa during hemorrhage and resuscitation. An experimental study in pigs. Eur Surg Res. (2009) 42:122–9. 10.1159/000193295 19155629

[43] Venkatesh B Morgan TJ Lipman J Subcutaneous oxygen tensions provide similar information to ileal luminal CO_2_ tensions in an animal model of haemorrhagic shock. Intens Care Med. (2000) 26:592–600. 10.1007/s001340051209 10923735

[44] de Bruijn HS Meijers C van der Ploeg-van den Heuvel A Sterenborg HJ Robinson DJ. Microscopic localisation of protoporphyrin IX in normal mouse skin after topical application of 5-aminolevulinic acid or methyl 5-aminolevulinate. J Photochem Photobiol B. (2008) 92:91–7. 10.1016/j.jphotobiol.2008.05.005 18571933

[45] Wefers Bettink MA Harms FA Dollee N Specht PAC Raat NJH Schoonderwoerd GC. Non-invasive versus *ex vivo* measurement of mitochondrial function in an endotoxemia model in rat: toward monitoring of mitochondrial therapy. Mitochondrion. (2020) 50:149–57. 10.1016/j.mito.2019.11.003 31770610

[46] Grote J. Gewebeatmung in Physiologie des Menschen. Berlin; Heidelberg; New York: Springer-Verlag (1997).

[47] Ortiz-Prado E Dunn JF Vasconez J Castillo D Viscor G. Partial pressure of oxygen in the human body: a general review. Am J Blood Res. (2019) 9:1–14. 30899601 PMC6420699

[48] Nakagawa S Johnson PCD Schielzeth H. The coefficient of determination R(2) and intra-class correlation coefficient from generalized linear mixed-effects models revisited and expanded. J R Soc Interface. (2017) 14:134. 10.1098/rsif.2017.0213 28904005 PMC5636267

[49] Bentley DJ Newell J Bishop D. Incremental exercise test design and analysis. Sports Med. (2007) 37:575–86. 10.2165/00007256-200737070-00002 17595153

[50] Meyer T Lucía A Earnest CP Kindermann W. A conceptual framework for performance diagnosis and training prescription from submaximal gas exchange parameters–theory and application. Int J Sports Med. (2005) 26:S38–48. 10.1055/s-2004-830514 15702455

[51] Roecker K Mayer F Striegel H Dickhuth HH. Increase characteristics of the cumulated excess-CO2 and the lactate concentration during exercise. Int J Sports Med. (2000) 21:419–23. 10.1055/s-2000-3836 10961517

[52] Heyward V Advanced Fitness Assessment and Exercise Prescription. 5th ed Champaign, IL: Human Kinetics (2006).

[53] Boone J Koppo K Bouckaert J. The VO_2_ response to submaximal ramp cycle exercise: Influence of ramp slope and training status. Respir Physiol Neurobiol. (2008) 161:291–7. 10.1016/j.resp.2008.03.008 18448396

[54] Zoll J Sanchez H N'Guessan B Ribera F Lampert E Bigard X. Physical activity changes the regulation of mitochondrial respiration in human skeletal muscle. J Physiol. (2002) 543:191–200. 10.1113/jphysiol.2002.019661 12181291 PMC2290497

[55] Gläser S Ittermann T Schäper C Obst A Dörr M Spielhagen T. The Study of Health in Pomerania (SHIP) reference values for cardiopulmonary exercise testing. Pneumologie. (2013) 67:58–63. 10.1055/s-0032-1325951 23247595

[56] Boone J Bourgois J. The oxygen uptake response to incremental ramp exercise: methodogical and physiological issues. Sports Med. (2012) 42:511–26. 10.2165/11599690-000000000-00000 22571502

[57] Hansen JE Casaburi R Cooper DM Wasserman K. Oxygen uptake as related to work rate increment during cycle ergometer exercise. Eur J Appl Physiol Occupat Physiol. (1988) 57:140–5. 10.1007/BF00640653 3349978

[58] Dempsey JA Wagner PD. Exercise-induced arterial hypoxemia. J Appl Physiol (1985). (1999) 87:1997–2006. 10.1152/jappl.1999.87.6.1997 10601141

