# Supplementary figures and images for: A Pilot Study on the Association of Mitochondrial Oxygen Metabolism and Gas Exchange During Cardiopulmonary Exercise Testing: Is There a Mitochondrial Threshold?
Source: Front Med (Lausanne). 2020 Dec 21;7:585462. doi: 10.3389/fmed.2020.585462 (PMC7779397; doi:10.3389/fmed.2020.585462)

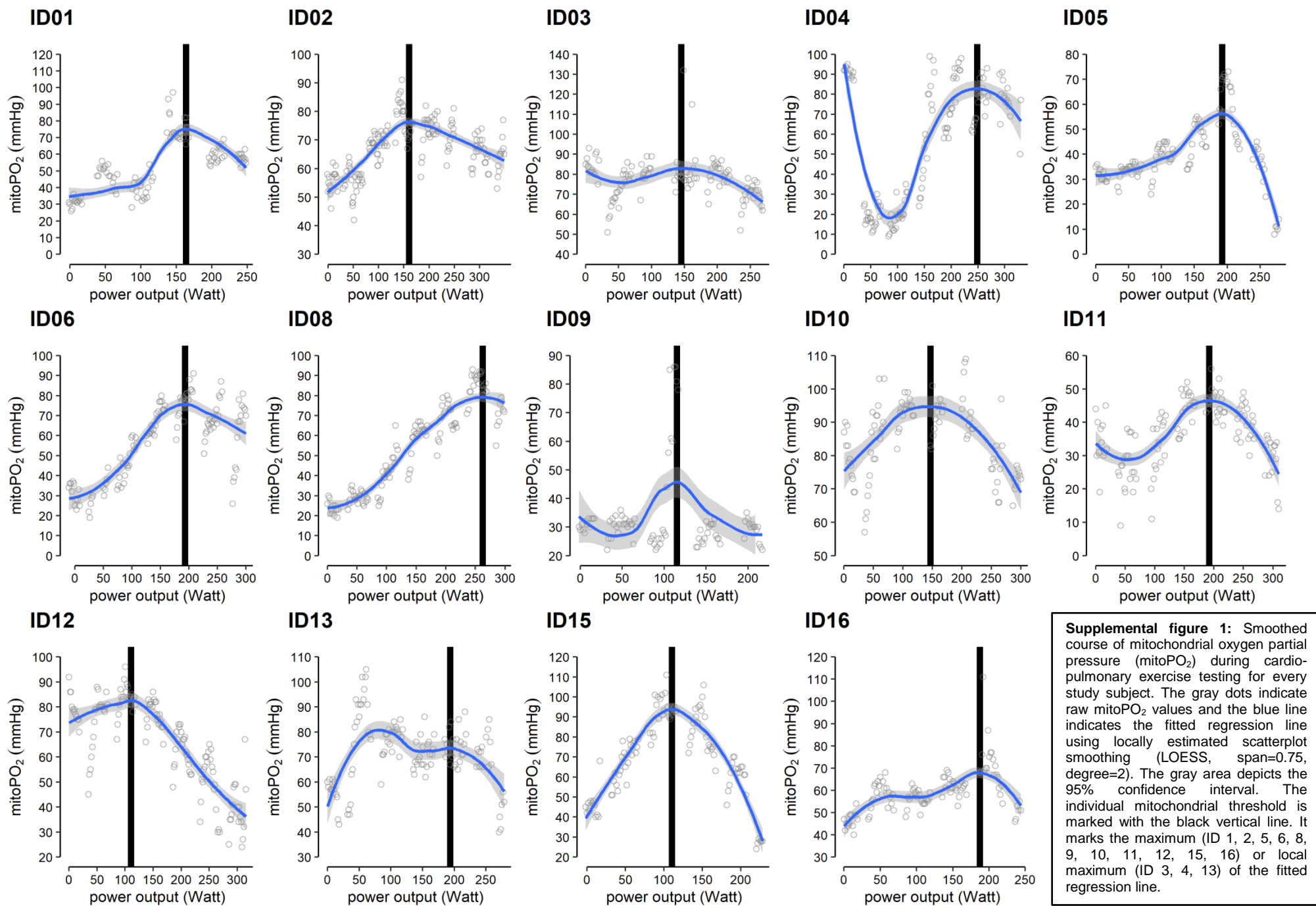

Supplement: Supplementary Figure 1 — Smoothed course of mitochondrial oxygen partial pressure (mitoPO2) during cardiopulmonary exercise testing for every study subject. The gray dots indicate raw mitoPO2 values and the blue line indicates the fitted regression line using locally estimated scatterplot smoothing (LOESS, span = 0.75, degree = 2). The gray area depicts the 95% confidence interval. The individual mitochondrial threshold is marked with the black vertical line. It marks the maximum (ID 1, 2, 5, 6, 8, 9, 10, 11, 12, 15, 16) or local maximum (ID 3, 4, 13) of the fitted regression line. [file Image_1.pdf]
