# Supplementary material for: A Pilot Study on the Association of Mitochondrial Oxygen Metabolism and Gas Exchange During Cardiopulmonary Exercise Testing: Is There a Mitochondrial Threshold?
Source: Front Med (Lausanne). 2020 Dec 21;7:585462. doi: 10.3389/fmed.2020.585462 (PMC7779397; doi:10.3389/fmed.2020.585462)
Supplement: Supplementary file 3 [file Data_Sheet_1.docx]

**Additional information for the reviewers**

| 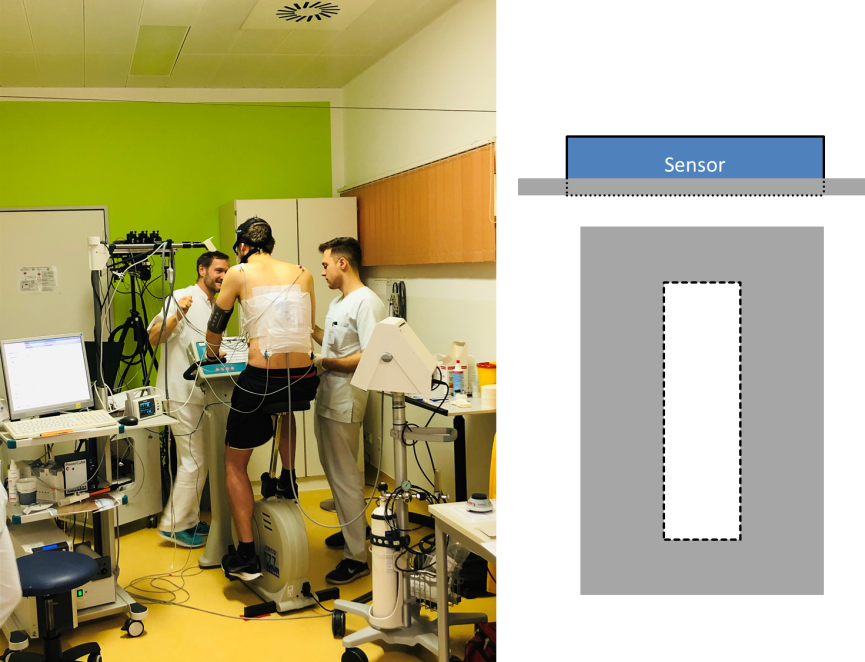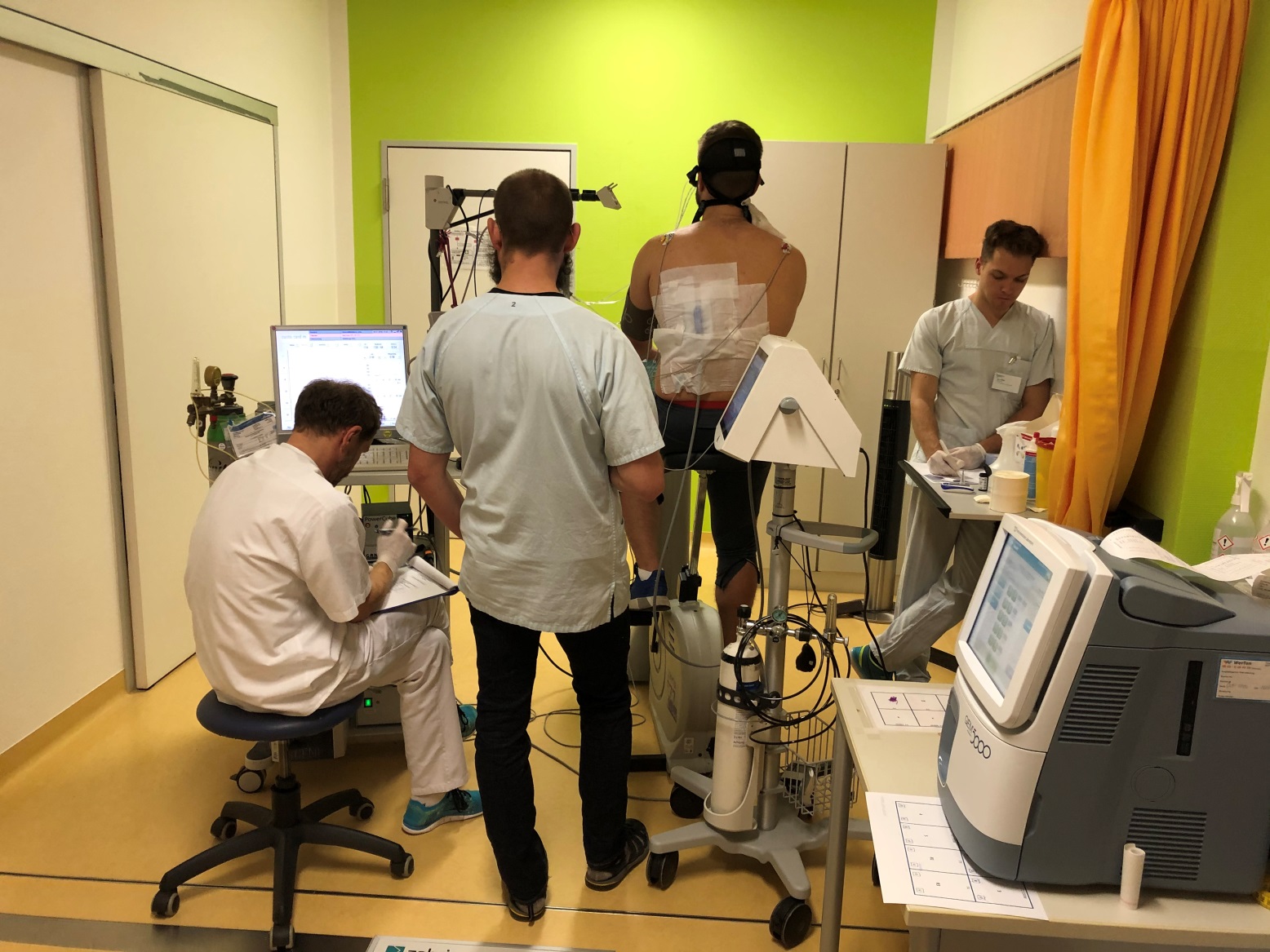 |
| --- |
| **Fig-R-1:** Experimental setup and fixation of the sensor at the back of the subject. The right part of the figure shows the sheath. The sensor was put in the notch to avoid sensor movements and to protect it from ambient light. |

**Table R-1:** Summary of intra-class correlation coefficients (ICC) and 95% confidence intervals (CI).

| **variable** | **type** | **ICC** | **95% CI** | | **p** |
| --- | --- | --- | --- | --- | --- |
|  |  |  | **lower** | **upper** |  |
| **mitoPO_2_** | *agreement* | 0.999 | 0.998 | 0.999 | <.0001 |
|  | *consistency* | 0.999 | 0.998 | 0.999 | <.0001 |
| **mitoVO_2:max_** | *agreement* | 0.955 | 0.937 | 0.968 | <.0001 |
|  | *consistency* | 0.955 | 0.937 | 0.968 | <.0001 |
| **mitoVO_2:avg_** | *agreement* | 0.955 | 0.937 | 0.968 | <.0001 |
|  | *consistency* | 0.955 | 0.937 | 0.968 | <.0001 |
| **mitoDO_2:max_** | *agreement* | 0.964 | 0.949 | 0.975 | <.0001 |
|  | *consistency* | 0.965 | 0.950 | 0.975 | <.0001 |
| **mitoDO_2:avg_** | *agreement* | 0.964 | 0.949 | 0.974 | <.0001 |
|  | *consistency* | 0.965 | 0.950 | 0.975 | <.0001 |
